# The Synthesis of L-Alanyl and β-Alanyl Derivatives of 2-Aminoacridone and Their Application in the Detection of Clinically-Important Microorganisms

**DOI:** 10.1371/journal.pone.0158378

**Published:** 2016-07-08

**Authors:** Marie Cellier, Arthur L. James, Sylvain Orenga, John D. Perry, Graeme Turnbull, Stephen P. Stanforth

**Affiliations:** 1 Research & Development Microbiology, bioMérieux SA, La-Balme-les-Grottes, France; 2 Department of Applied Sciences, Northumbria University, Newcastle upon Tyne, United Kingdom; 3 Department of Microbiology, Freeman Hospital, Newcastle upon Tyne, United Kingdom; University of Gdansk, POLAND

## Abstract

In clinical microbiology the speed with which pathogenic microorganisms may be detected has a direct impact on patient health. One important strategy used in the laboratory is the growth of cultures in the presence of an enzymatic substrate which, once transformed by the appropriate microbial enzyme, generates a detectable colour or fluorescence output. Such substrates have previously been prepared by our group and others and are available as commercial diagnostic kits, however they all suffer from some degree of diffusion when used in a solid growth medium. This diffusion complicates the detection and differentiation of species in polymicrobial cultures and so we sought to improve on our previous work. In this work we have prepared and evaluated a series of novel fluorogenic enzyme substrates based on *N*-substituted-2-aminoacridones. All of the prepared substrates were found to be suitable for the detection and differentiation of certain microorganisms, however those based on the 2-amino-10-benzylacridone core in particular showed no apparent diffusion when incorporated into solid growth media. On transformation these substrates generated brightly fluorescent colonies that are clearly contrasted with the background medium due to the difference in emission wavelength (λ_em_ 445–450 nm for the substrate, λ_em_ 550 nm for the product). Here we have shown that our L-alanyl aminopeptidase substrate, 2-(*N*-L-alanylamino)-10-benzylacridone, is particularly suited to the detection of Gram-negative bacteria, and our β-alanyl aminopeptidase substrate, 2-(*N*- β-alanylamino)-10-benzylacridone, to the detection of *Pseudomonas aeruginosa* and *Serratia marcescens* when grown on solid media incorporating these substrates. The resulting fluorophore shows no apparent diffusion from the colonies of interest, and the enhanced sensitivity offered by fluorescent emission may allow for the detection of these organisms as microcolonies using automated fluorescence microscopy.

## Introduction

The use of enzymatic substrates as tools for the detection and identification of clinically-important microorganisms is a subject of particular interest in the health-care sector [1–3]. In diagnostic microbiology, the detection of specific types of aminopeptidase activities has proved useful. Thus, Gram-positive and Gram-negative microorganisms can often be differentiated by their L-alanine aminopeptidase activities [4,5]. This enzyme is ubiquitous in Gram-negative microorganisms whereas it is usually absent from most Gram-positive microorganisms. β-Alanyl aminopeptidase has been detected in *Pseudomonas aeruginosa*, a common respiratory pathogen in cystic fibrosis patients [6].

**Fig 1:**
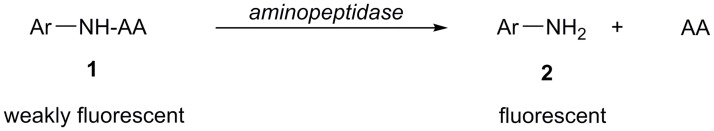
Fluorescence generation from aminopeptidase activity (AA = amino acid).

One important strategy that has been utilised for microorganism detection and identification is to grow the microorganisms in the presence of an enzymatic substrate; microorganisms which possess the appropriate enzymes can then transform the substrate into a detectable product. The release of highly fluorescent heterocyclic amines **2** from weakly fluorescent enzyme substrates **1** possessing a heterocyclic moiety has been employed as a protocol for detecting aminopeptidase activities, as illustrated in Fig 1.

**Fig 2:**
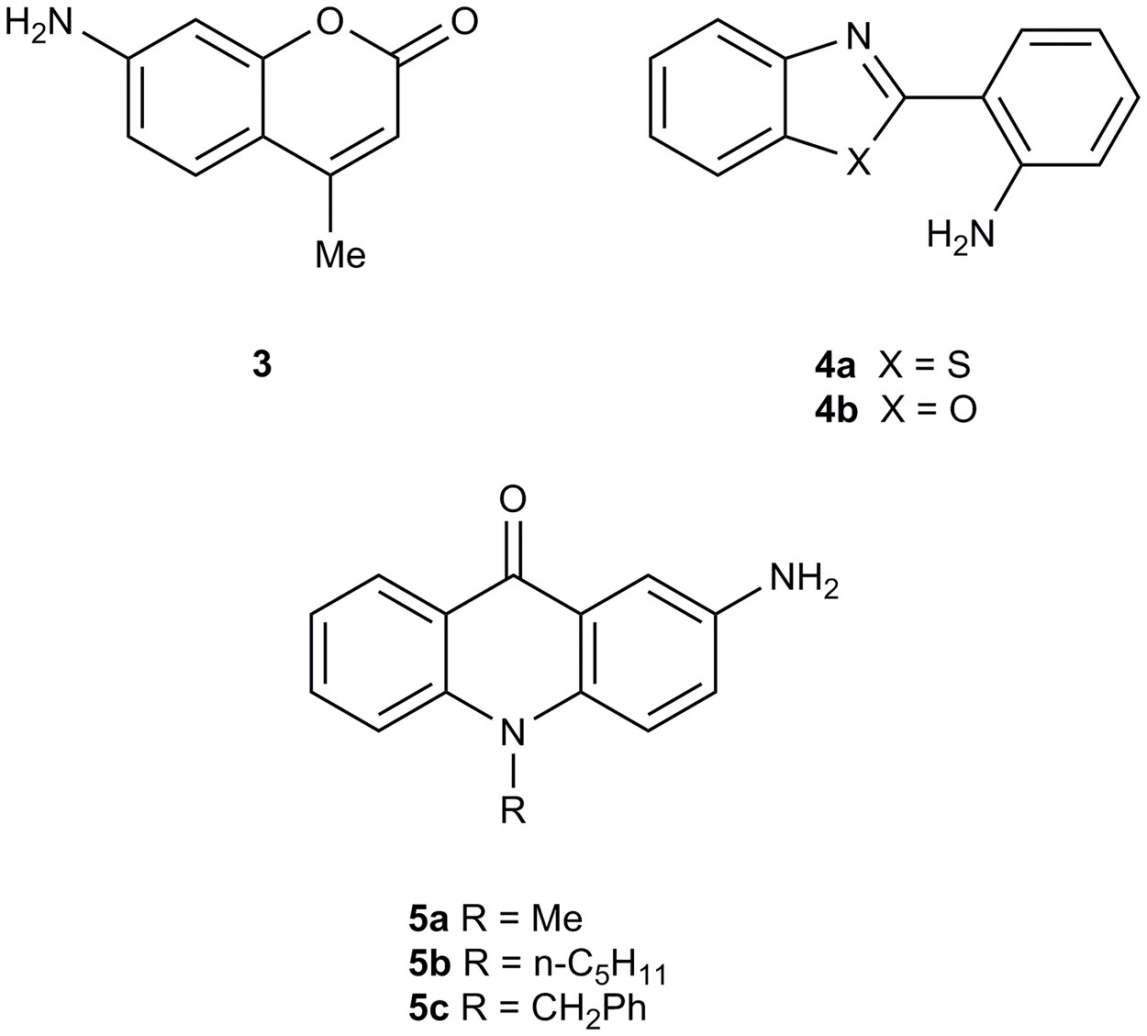
Fluorescent heterocyclic amines as core molecules for the preparation of fluorogenic aminopeptidase substrates.

Substrates of 7-amino-4-methylcoumarin **3** are currently used in commercial diagnostic kits, and we have previously described the synthesis of fluorogenic L-alanyl and β-alanyl aminopeptidase substrates derived from 2-(2-aminophenyl)benzothiazoles **4a** and 2-(2-aminophenyl)benzoxazoles **4b** and their evaluation against a range of clinically-important microorganisms (Fig 2) [7–9] Each of these substrates generate fluorophores which are successful in detecting and differentiating microorganisms based on their aminopeptidase activity, however they all suffer from some degree of diffusion of the fluorophore into the surrounding growth medium. Most microbiological cultures, whether derived from the culture of food, clinical or environmental samples are polymicrobial in nature (i.e. they contain more than one species), and diffusion of a liberated fluorophore into the media can reduce the sensitivity of the test. In this communication we report the synthesis and initial evaluation of L-alanyl and β-alanyl aminopeptidase substrates derived from *N*-substituted-2-aminoacridones **5a-c**. These generate a highly fluorescent product which is yellow in colour (reducing the impact of cellular autofluorescence) and which does not appear to diffuse (providing high contrast to the growth medium).

## Methods

**Fig 3:**
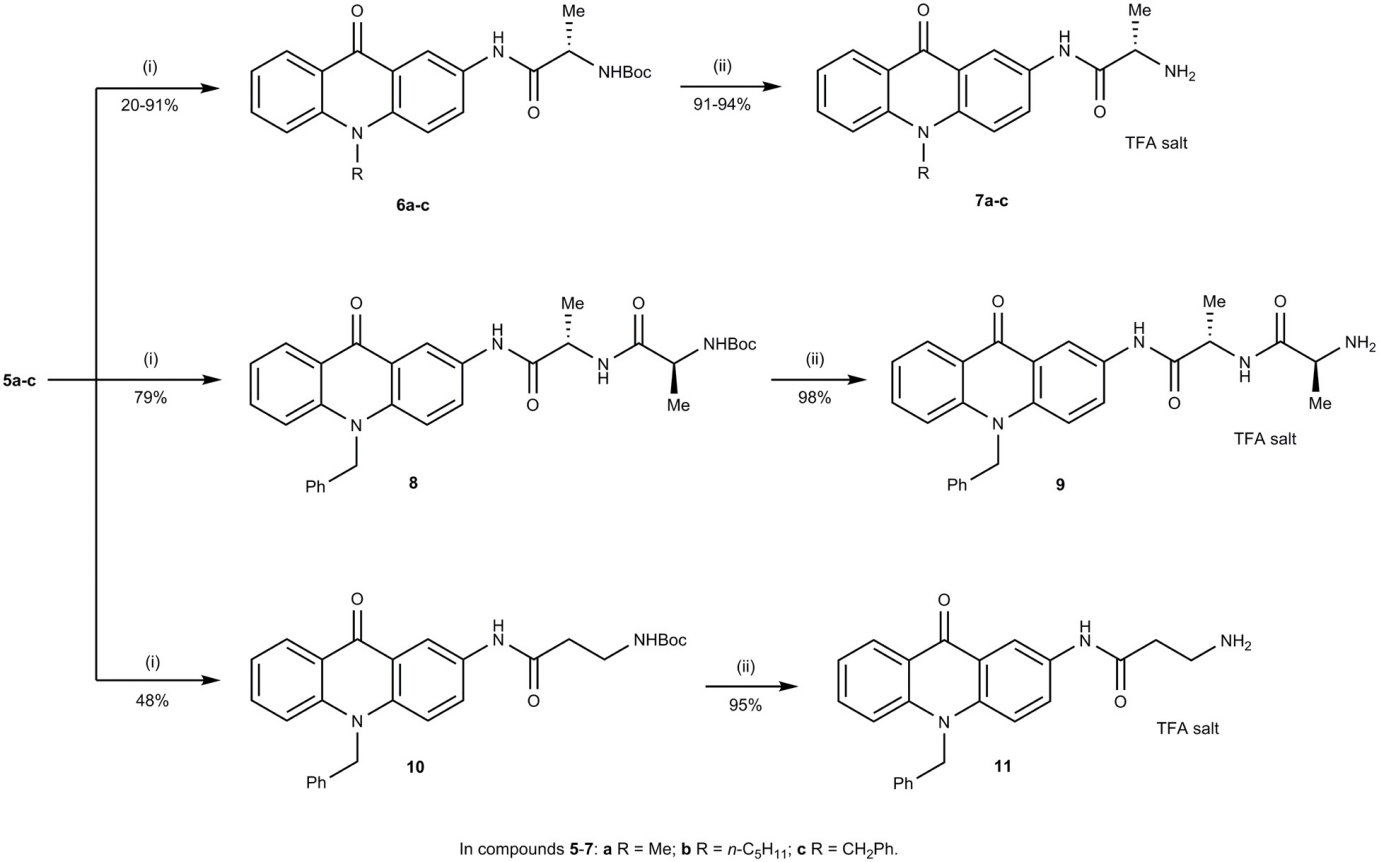
Synthesis of aminopeptidase substrates. Reagents and conditions: (i) *N*-Boc-protected amino acid, *N*-methyl morpholine, isobutyl chloroformate, anhydrous THF, -12°C to RT overnight; (ii) TFA, RT, 2 h.

The required core amines **5a-c** were readily prepared by alkylation of 2-nitroacridone [10] under basic conditions followed by reduction of the nitro-group (see ESI for synthetic procedures and characterisation data). The 2-aminoacridones **5a-c** were converted into their corresponding Boc-protected amino acid derivatives **6a-c**, **8** and **10** as depicted in Fig 3. Deprotection of these Boc-protected compounds using trifluoroacetic acid afforded the desired aminopeptidase substrates **7a-c**, **9** and **11** respectively.

**Fig 4:**
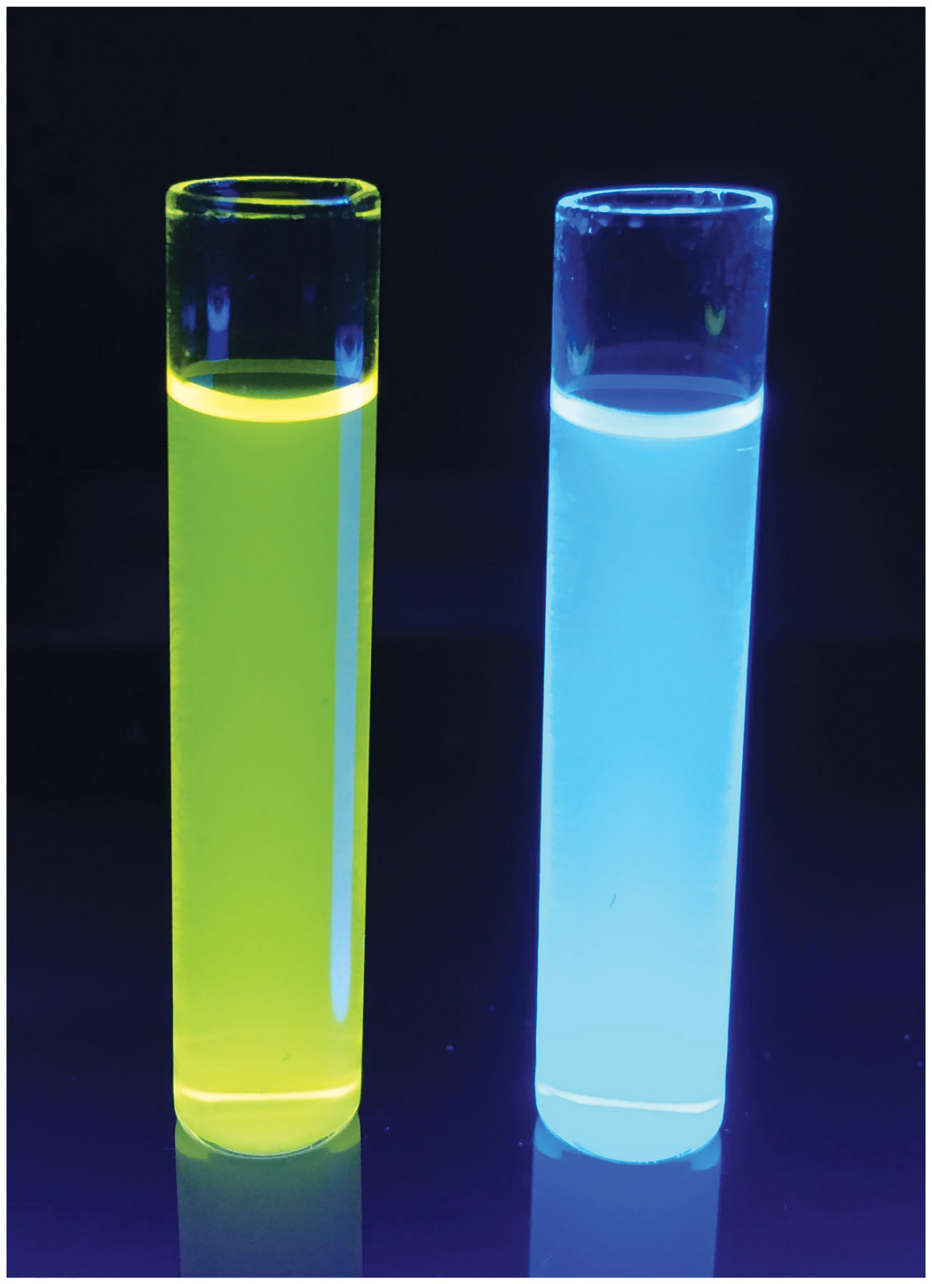
Solutions of amine 5c (left) and substrate 7c (right) in ethanol viewed under UV illumination at 365 nm (both at 10 μM).

The fluorescence excitation and emission wavelengths for the core amines **5a-c** and the substrates **7a-c**, **9** and **11** in ethanol are collected in Table 1. Solutions of the core amines **5a-c** all displayed yellow fluorescence (λ_em_ 545–546 nm). Conjugation through the 2-amino group significantly reduces the Stokes’ shift of the emission, and solutions of the substrates **7a-c**, **9** and **11** showed blue fluorescence (λ_em_ 445–453 nm). Fig 4 depicts ethanolic solutions of amine **5c** and its corresponding L-alanyl aminopeptidase substrate **7c** when viewed under UV illumination at 365 nm.

**Table 1:** Excitation and emission wavelengths of amines 5a-c and substrates derived from these amines in ethanol solution (all 1 mg L^-1^).

Amine	λ_ex_	λ_em_	Substrate	λ_ex_	λ_em_
**5a**	434^a^	546	**7a**	418	453
**5b**	435	545	**7b**	401,418	445
**5c**	429	550	**7c**	415	445
			**9**	414	450
			**11**	414	449

^a^lit., 438 nm (EtOH).

## Microbiological Evaluation and Discussion

The substrates **7a-c**, **9** and **11** were evaluated in Columbia agar medium against a panel of clinically-important microorganisms including 10 Gram-negative bacteria, 8 Gram-positive bacteria and 2 yeasts (see ESI for microbiological procedures). The growth of the microorganisms after incubation (18 h, 37°C) was compared to a control in which no substrate was present. Table 2 depicts the extent of growth of the various Gram-negative microorganisms and the fluorescence generated by hydrolysis of the substrates by L-alanyl and β-alanyl aminopeptidases (full results are supplied in the ESI). The substrates were all evaluated at a concentration of 50 mg L^-1^ and the *N*-benzylated, L-alanyl substrate **7c** was also evaluated at a concentration of 100 mg L^-1^.

**Table 2:** Growth and fluorescence of Gram-negative microorganisms in the presence of substrates 7a-c.

			Substrate					
		Control	7a		7b		7c	
	Microorganism / Reference^a^	Growth^b^	Growth^b^	Fluorescence^c^	Growth^b^	Fluorescence^c^	Growth^b^	Fluorescence^c^
1	*Escherichia coli* NCTC 10418	++	-	-	-	-	++	++ yellow
2	*Klebsiella pneumoniae* NCTC 9528	++	-	-	-	-	++	-
3	*Providencia rettgeri* NCTC 7475	++	++	++ yellow	++	+ yellow	++	++ yellow
4	*Enterobacter cloacae* NCTC 11936	++	++	++ yellow	++	+ yellow	++	++ yellow
5	*Serratia marcescens* NCTC 10211	++	++	++ yellow	++	++ yellow	++	++ yellow
6	*Salmonella typhimurium* NCTC 74	++	++	++ yellow	++	+ yellow	++	+/- yellow
7	*Pseudomonas aeruginosa* NCTC 10662	++	++	++ yellow	++	+ yellow	++	++ yellow
8	*Yersinia enterocolitica* NCTC 11176	++	++	+ yellow	++	+ yellow	++	+ yellow
9	*Burkholderia cepacia* NCTC 10743	++	++	++ yellow	++	+ blue	++	++ blue
10	*Acinetobacter baumannii* NCTC 12156	++	++	++ yellow	-	-	+	+ yellow
	Background		-	+ blue	-	+ blue	-	+ blue

^a^ NCTC: National Collection of Type Cultures; ATCC: American Type Culture Collection; NCPF: National Collection of Pathogenic Fungi.

^b^ ++ strong growth, + moderate growth,—no growth.

^c^ ++ strong fluorescence, + moderate fluorescence, +/- weak fluorescence,—no fluorescence.

In the control, Gram-negative microorganisms exhibited strong growth (all graded ++) whereas Gram-positive microorganisms and yeasts only displayed moderate growth (all graded +). The majority of Gram-negative microorganisms exhibited strong growth in the presence of the substrates when the substrate concentration was 50 mg L^-1^, with the exception of *Escherichia coli* and *Klebsiella pneumoniae* which did not grow in the presence of substrates **7a** and **7b**. The growth of the Gram-positive microorganisms was frequently diminished in comparison with the control, indicating an inhibitory effect of some of the substrates on some of these bacteria. There was a clear inhibitory effect on all of the microorganisms by substrate **7c** at a concentration of 100 mg L^-1^ in comparison with the growth observed at a concentration of 50 mg L^-1^.

**Fig 5:**
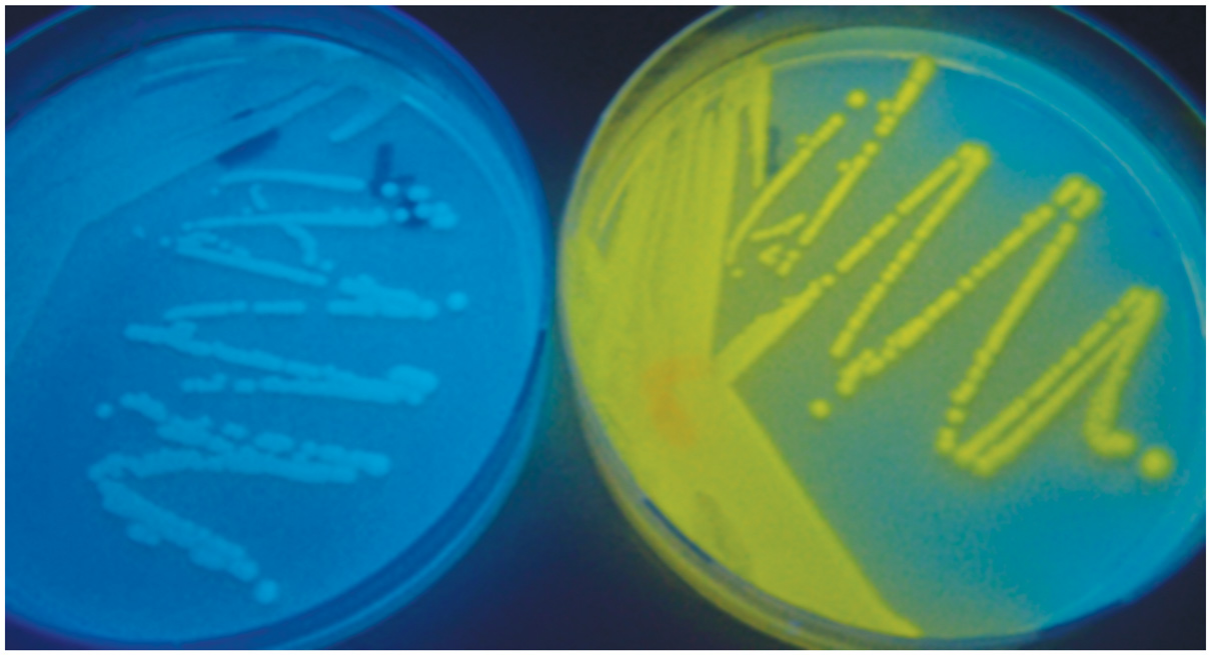
Colonies of *S*. *aureus* (left) and *E*. *coli* (right) after incubation in the presence of substrate 7c.

All of the L-alanyl aminopeptidase substrates were able to produce bright yellow fluorescent colonies with most Gram-negative bacteria when viewed under UV light (365 nm), indicating hydrolysis of the substrate had occurred. The background fluorescence was blue as a consequence of the blue fluorescence of the unreacted substrate but there was a clear contrast between the yellow and blue fluorescence of the colonies and the background respectively. This is illustrated in Fig 5. Enzyme substrates for microorganism detection are generally designed to be weakly fluorescent or non-fluorescent, only producing strong fluorescence after release of the core fluorophore, however the fluorescence of both substrate and product in this work is not a disadvantage because of their different emission wavelengths.

Substrate **7a** gave strongly fluorescent yellow colonies with most Gram-negative microorganisms. Neither the Gram-positive microorganisms nor the yeasts produced fluorescent colonies with this substrate. The activity of substrate **7b** was broadly similar to substrate **7a**, but the fluorescence observed with the Gram-negative microorganisms was generally weaker. Blue fluorescent colonies were seen with *Burkholderia cepacia* and this was attributed to concentration of the substrate within the colonies without hydrolysis occurring. Substrate **7c** gave fluorescent yellow colonies of variable intensities with the majority of the Gram-negative microorganisms, and weak, yellow fluorescent colonies with two of the Gram-positive microorganisms. At a higher substrate concentration (100 mg L^-1^), this substrate was more inhibitory to microorganism growth and hence was observed to be more selective with six of the ten Gram-negative producing weakly fluorescent colonies. Good microorganism growth was seen with the di-L-alanyl substrate **9** with the majority of the Gram-negative microorganisms producing yellow fluorescent colonies of variable intensities.

As anticipated, the β-alanyl aminopeptidase substrate **11** gave intense, yellow fluorescent colonies with *Pseudomonas aeruginosa*. This substrate was also effective at detecting *Serratia marcescens*, a microorganism that is also known to possess β-alanyl aminopeptidase activity [4].

Each of these substrates are potentially useful for microbiological applications as they are cleaved to generate brightly fluorescent products that are clearly contrasted with the background culture medium due to their differing fluorescence emission wavelengths. Moreover, the fluorescent product released by hydrolysis remains restricted to the bacterial colonies and does not appear to diffuse through the agar. This lack of diffusion means that a colony expressing a specific aminopeptidase may be differentiated from a neighbouring colony that does not express this activity using these substrates, such as in polymicrobial cultures routinely generated from food and clinical samples. These combined attributes make these substrates a useful alternative to many conventional substrates such as those based on 7-amino-4-methylcoumarin, which emits light in the blue region of the spectrum [11,12] and can suffer from a reduction in sensitivity by cellular autofluorescence.

Chromogenic culture media are widely used for detection of bacterial pathogens as they offer high specificity by targeting specific enzyme activities using chromogenic enzyme substrates. (e.g. ref. [13]). For example, the β-alanyl aminopeptidase activity of *Pseudomonas aeruginosa* has been targeted in a chromogenic culture medium for detection of this important pathogen [14]. Fluorescent substrates such as **11** may allow a similar approach with the generation of a fluorescent (rather than a chromogenic) product, and these are currently not widely used in solid media. The enhanced sensitivity offered by fluorescence and high-contrast afforded by these substrates may allow for the specific detection of pathogenic bacteria with reduced time-to-detection using automated detection of fluorescent microcolonies before they are visible to the naked eye [15].

## Supporting Information

S1 File Supporting information. (DOCX)
