# Supplementary material for: The Synthesis of L-Alanyl and β-Alanyl Derivatives of 2-Aminoacridone and Their Application in the Detection of Clinically-Important Microorganisms
Source: PLoS One. 2016 Jul 8;11(7):e0158378. doi: 10.1371/journal.pone.0158378 (PMC4938530; doi:10.1371/journal.pone.0158378)
Supplement: S1 File — (DOCX) [file pone.0158378.s001.docx]

**Supplementary Information**

**The synthesis of L-alanyl and β-alanyl derivatives of 2-aminoacridone and their application in
the detection of clinically-important microorganisms.**

Marie Cellier, the late Arthur L. James, Sylvain Orenga, John D. Perry,
Graeme Turnbull and Stephen P. Stanforth.

Contents

[1. Detailed experimental procedures. 3](#_Toc436399858)

[1.1 Synthesis of 2-nitroacridone (2-nitro-10*H*-acridin-9-one). 4](#_Toc436399859)

[1.2 Synthesis of *N*-substituted-2-nitroacridones. 4](#_Toc436399860)

[1.2.1 10-Methyl-2-nitroacridone. 4](#_Toc436399861)

[1.2.2 2-Nitro-10-pentylacridone. 5](#_Toc436399862)

[1.2.3 10-Benzyl-2-nitroacridone. 5](#_Toc436399863)

[1.3 General procedure for the preparation of *N*-substituted-2-aminoacridones. 6](#_Toc436399864)

[1.3.1 2-Amino-10-methylacridone, 5a 6](#_Toc436399865)

[1.3.2 2-Amino-10-pentylacridone, 5b 6](#_Toc436399866)

[1.3.3 2-Amino-10-benzylacridone, 5c 7](#_Toc436399867)

[1.4 General procedure for the preparation of Boc-protected amino acid derivatives of *N*-substituted-2-aminoacridones. 7](#_Toc436399868)

[1.4.1 2-(Boc-L-alanylamino)-10-methylacridone, 6a 8](#_Toc436399869)

[1.4.2 2-(Boc-L-alanylamino)-10-pentylacridone, 6b 8](#_Toc436399870)

[1.4.3 2-(Boc-L-alanylamino)-10-benzylacridone, 6c 9](#_Toc436399871)

[1.4.4 2-(Boc-L-alanyl-L-alanylamino)-10-benzylacridone, 8 9](#_Toc436399872)

[1.4.5 2-(Boc-β-alanylamino)-10-benzylacridone, 10 10](#_Toc436399873)

[1.5 General procedure for the deprotection of Boc-protected amino acid derivatives of *N*-substituted 2-aminoacridones. 11](#_Toc436399874)

[1.5.1 2-(L-Alanylamino)-10-methylacridone TFA salt, 7a 11](#_Toc436399875)

[1.5.2 2-(L-Alanylamino)-10-pentylacridone TFA salt, 7b 11](#_Toc436399876)

[1.5.3 2-(L-Alanylamino)-10-benzylacridone TFA salt, 7c 12](#_Toc436399877)

[1.5.4 2-(L-Alanyl-L-alanylamino)-10-benzylacridone TFA salt, 9 12](#_Toc436399878)

[1.5.5 2-(β-Alanylamino)-10-benzylacridone, 11 13](#_Toc436399879)

[2. Microbiological screening procedure. 14](#_Toc436399880)

[3. Full tables of microorganism screening results. 15](#_Toc436399881)

[4. References 18](#_Toc436399882)

[Appendix 19](#_Toc436399883)

# 1. Detailed experimental procedures.

**NMR spectra** were recorded as indicated on either a JEOL EX270 Delta spectrometer at frequencies of 270.17 MHz for ^1^H NMR and 67.93 MHz for ^13^C NMR, or on a JEOL ECS400 Delta spectrometer at frequencies of 399.78 MHz for ^1^H NMR and 100.53 MHz for ^13^C NMR. All chemical shifts are quoted as parts per million (ppm) relative to tetramethylsilane (TMS) as an internal standard in either deuterated dimethyl sulfoxide (DMSO-*d_6_*) or deuterated chloroform (CDCl_3_). Shifts are reported as follows: Shift (*δ*) in ppm (multiplicity, coupling constant in Hz, normalised integral, assignment). Multiplicities of signals are expressed as: s = singlet, d = doublet, t = triplet, q = quartet,
quint = quintet, sex = sextet, m = multiplet, and in combinations thereof.

**Low-resolution mass spectra** were recorded *via* direct injection of dilute methanolic solutions (containing 0.1% formic acid) into a Thermo Finnigan LCQ Advantage MS detector using electrospray ionisation (ESI).

**High-resolution mass spectra (HRMS)** were obtained from the EPSRC National Mass Spectrometry Service, Swansea University. Spectra were recorded using a Finnigan MAT 900 XLT high-resolution double focussing mass spectrometer using nano-electrospray ionisation (NSI).

**Melting points** were recorded in open capillary tubes using a Stanford Research Systems MPA160 Melting Point Apparatus and are reported uncorrected.

**Infra-red spectra** were recorded *via* a SensIR Technologies Durascope diamond anvil cell mounted on a Perkin-Elmer Paragon 1000FT-IR Spectrometer. Wavenumbers are reported in cm^-1^ [wavenumber(intensity)]. Intensities are expressed as: w = weak, m = medium, s = strong.

**Ultraviolet spectra** were recorded as dilute ethanolic solutions *via* a Varian Cary 50 Bio UV/Visible Spectrophotometer.

**Fluorescence spectra** were recorded as dilute ethanolic solutions *via* a Thermo Spectronic Amino-Bowman Series 2 luminescence spectrometer.

**Thin-layer chromatography** was performed on Merck plastic foil plates pre-coated with silica gel 60 F_254_.

**Column chromatography** was performed using Fisher Scientific silica 60A (35-70 μm).

**Reagents** were purchased from Sigma-Aldrich, Lancaster, Alfa Aesar, BDH Chemicals and Apollo Scientific and were used without further purification unless otherwise noted.

**Solvents** were obtained from Fisher Scientific and were of either Reagent or HPLC Grade. Solvents were dried as required over activated 4Å molecular sieves.

## Synthesis of 2-nitroacridone (2-nitro-10*H*-acridin-9-one).

Acridone (19.50 g, 100 mmol) was stirred in aqueous AcOH (35%, 75 mL) and a mixture of AcOH (150 mL) and HNO_3_ (78%, 75 mL) was added slowly before heating the mixture to 55 ºC. After 4 h the mixture was cooled and poured onto ice (600 g) with stirring. The resulting yellow precipitate was collected and rinsed with water. The filter cake was broken up and twice stirred in hot AcOH (500 mL) to dissolve the unwanted 4-nitro isomer. The crude product was recrystallised from aqueous DMF to yield 2-nitroacridone (13.35 g, 56%) as a yellow powder.

## Synthesis of *N*-substituted-2-nitroacridones.

### 10-Methyl-2-nitroacridone.

2-Nitroacridone (0.96 g, 4.0 mmol) was added to dry DMF (20 mL) with stirring at RT under a N_2_ atmosphere. NaH (0.48 g, 12.0 mmol as a 60% mixture in mineral oil) was added and the mixture stirred at 45 ºC. After 45 min the mixture was cooled in an ice-water bath and methyl iodide (0.75 mL, 12.0 mmol) was added. After 30 min the mixture was heated to 85 ºC for 4 h before cooling to room temperature. Water (10 mL) was added and the precipitate filtered and resuspended in Et_2_O (40 mL) to remove residual DMF. The solid was filtered and recrystallized from aqueous DMF to yield 2-nitro-10-methylacridone (0.40 g, 39%) as a brown crystalline powder.
*δ*_H_ (270 MHz; DMSO-*d_6_*) 9.02 (d, *J* = 2.9 Hz, 1H, Ar-H), 8.51 (dd, *J* = 9.4 Hz, *J* = 2.9 Hz, 1H, Ar-H), 8.33 (dd,
*J* = 7.8 Hz, *J* = 1.7 Hz, 1H, Ar-H), 8.04 (d, *J* = 9.4 Hz, 1H, Ar-H), 7.96-7.87 (m, 2H, 2 x Ar-H), 7.46 (ddd, *J* = 7.8 Hz, *J* = 6.2, *J* = 2.0 Hz, 1H, Ar-H), 3.99 (s, 3H, CH_3_). MS (+ESI) *m/z* 255.1 ([M+H]^+^; 32%). M.Pt. >254 ºC (lit. 282.9-284.0 ºC)^1^. ν_max­_/cm^-1^ 3076w, 1684m, 1601s, 1321s, 1262s, 1172m, 1080m, 745s, 671.

### 2-Nitro-10-pentylacridone.

2-Nitroacridone (1.00 g, 4.16 mmol) was added to dry DMF (50 mL) under a CaCl_2_ trap with stirring at RT. NaH (0.50 g as a 60% dispersion in mineral oil, 12.50 mmol) was added and the mixture stirred at 45 ºC. After 45 min the mix was cooled in an ice-water bath and pentyl bromide (1.55 mL, 12.47 mmol) was added. The mixture was heated at 90 ºC for 18 h before evaporating the solvent. Ice (40 g) was added to the residue and the resulting orange precipitate collected, rinsed with Et_2_O, and dried in air. The crude product (1.22 g) was found to be a mixture of
2-nitro-10-pentylacridone and 2-nitroacridone by ^1^H NMR spectroscopy in 2:1 ratio (as determined by peak integrals), however separation by column chromatography proved difficult. The compound was purified after reduction to
2-amino-10-pentylacridone. ^1^H NMR signals corresponding to 2-nitro-10-pentylacridone were as follows: *δ*_H_ (400 MHz; DMSO-*d_6_*) 8.97 (d, *J* = 3.2 Hz, 1H, Ar-H), 8.45 (dd, *J* = 9.6 Hz, *J* = 3.2 Hz, 1H, Ar-H), 8.30 (dd, *J* = 7.3 Hz,
*J* = 1.4 Hz, 1H, Ar-H), 7.95 (d, *J* = 9.6 Hz, 1H, Ar-H), 7.88-7.86 (m, 2H, 2 x Ar-H), 7.40 (ddd, *J* = 7.8 Hz, *J* = 6.0 Hz, *J* = 1.8 Hz, 1H, Ar-H), 4.46 (t, *J* = 7.8 Hz, 2H, NCH_2_), 1.80-1.72 (m, 2H, CH­_2_), 1.50-1.43 (m, 2H, CH_2_), 1.40-1.31 (m, 2H, CH_2_), 0.97 (t, *J* = 7.3 Hz, 3H, CH_3_). MS (+ESI) 311.1 ([M+H]^+^; 100%), 2-nitroacridone not detected. ν_max_/cm^-1^ 3098w, 2927w, 2858w, 1603s, 1473s, 1325s, 1170m, 1081m, 760s, 746s, 673d.

### 10-Benzyl-2-nitroacridone.

2-Nitroacridone (2.40 g, 10.0 mmol) was added to dry DMF (50 mL) under a CaCl­_2_ trap with stirring at RT. NaH (1.20 g, 50.0 mmol as a 60% dispersion in mineral oil) was added and the mixture stirred at 45 ºC. After 45 min the mix was cooled in an ice-water bath and benzyl bromide (3.56 mL, 29.9 mmol) was added. After 30 min, the mixture was heated to 90 ºC for 4 h before pouring onto ice. The resulting red precipitate was filtered and the filter cake washed with Et_2_O. The crude product was recrystallised from AcOH to yield 10-benzyl-2-nitroacridone (3.09 g, 94%) as an orange crystalline powder. *δ*_H_ (400 MHz; DMSO-*d_6_*) 9.10 (d, *J* = 2.8 Hz, 1H, H1), 8.50 (dd, *J* = 9.2 Hz,
*J* = 2.8 Hz, 1H, H3), 8.41 (dd, *J* = 7.9 Hz, *J* = 1.8 Hz, 1H, H8), 7.88-7.82 (m, 2H, H5 and H6), 7.73 (d, *J* = 9.2 Hz, 1H, H4), 7.49 (ddd, *J* = 7.9 Hz, *J* = 6.9 Hz, *J* = 0.9 Hz, 1H, H7), 7.38-7.27 (m, 5H, 5xAr-H), 5.91 (s, 2H, CH_2_).
*δ*_C_ (101 MHz; DMSO-*d_6_*) 176.8 (C=O), 146.2 (Ar-C), 142.4 (Ar-C), 141.5 (Ar-C), 135.9 (Ar-C), 135.8 (Ar-C), 129.5 (Ar-C), 128.5 (Ar-C), 128.1 (Ar-C), 127.3 (Ar-C), 126.3 (Ar-C), 123.8 (Ar-C), 123.5 (Ar-C), 122.5 (Ar-C), 121.2
(Ar-C), 118.6 (Ar-C), 117.5 (Ar-C), 50.3 (CH_2_). MS (+ESI) 682.7 ([2M+Na]^+^; 100%). M.Pt. 243.1-244.1 ºC (lit.
240 ºC)^2^. ν_max­_/cm^-1^ 3066w, 1644m, 1602s, 1475m, 1326m, 1279s, 1171s, 823s, 757s, 746s, 672s.

## General procedure for the preparation of *N*-substituted-2-aminoacridones.

*N*-Substituted-2-nitroacridone (1 eq.) and SnCl_2_·2H_2_O (2.5 eq.) were dissolved in EtOH and heated to reflux under a N_2_ atmosphere. After 4 h the solvent was evaporated and replaced by a 1:1 mixture of EtOAc and aqueous NaOH (4M). The mixture was stirred vigorously for 4 h before the resulting white precipitate was removed by filtration. The organic layer of the filtrate was separated and the aqueous layer extracted into EtOAc. The combined organic layers were washed with brine, dried over MgSO_4_ and then the solvent evaporated to yield the amine product.

### 2-Amino-10-methylacridone, 5a

Compound **5a** was prepared from 10-methyl-2-nitroacridone (215 mg, 0.85 mmol) and obtained as a yellow powder (184 mg, 97%) which was used without further purification. *δ*_H_ (400 MHz; DMSO-*d_6_*) 8.25 (d, *J* = 7.8 Hz, 1H, Ar-H), 7.73-7.66 (m, 2H, 2xAr-H), 7.61 (d, *J* = 9.2 Hz, 1H, Ar-H), 7.46 (d, *J* = 2.8 Hz, 1H, Ar-H), 7.20-7.15 (m, 2H, 2xAr-H), 5.25 (s, 2H, NH_2_), 3.84 (s, 3H, CH_3_). *δ*_C_ (101 MHz; DMSO-*d_6_*) 176.5 (C=O), 144.0 (Ar-C), 142.1 (Ar-C), 134.9 (Ar-C), 133.6 (Ar-C), 127.1 (Ar-C), 123.7 (Ar-C), 123.5 (Ar-C), 121.0 (Ar-C), 120.4 (Ar-C), 117.5 (Ar-C), 116.2 (Ar-C), 107.6 (Ar-C), 33.9 (CH_3_). MS (+ESI) *m/z* 225.1 ([M+H]^+^; 100%). M.Pt. 195.6-196.5 ºC (lit. 205 ºC)^3^. ν_max­_/cm^-1^ 3500-3200 m (broad), 2922m, 1613m, 1586s, 1552s, 1503s, 1462s, 1360m, 1177m, 755s. λ_EX_ = 434 nm (lit. 438 nm in EtOH)^4^, λ_EM_ = 546 nm (EtOH).

### 2-Amino-10-pentylacridone, 5b

Compound **5b** was prepared from 2-nitro-10-pentylacridone (1.09 g, 3.51 mmol). The crude product was purified by column chromatography (SiO_2_, 50-75% EtOAc in petroleum ether [60-80 ºC fraction]) to yield compound **5b** as a red crystalline powder (0.30 g, 29%). *δ*_H_ (400 MHz; DMSO-*d_6_*) 8.31 (dd, *J* = 7.8 Hz, *J* = 1.4 Hz, 1H, Ar-H), 7.77-7.71 (m, 2H, 2 x Ar-H), 7.61 (d, *J* = 9.2 Hz, 1H, Ar-H), 7.49 (d, *J* = 2.8 Hz, 1H, Ar-H), 7.24-7.20 (m, 2H, 2 x Ar-H), 5.29 (s, 2H, NH_2_), 4.41 (t, *J* = 8.2 Hz, 2H, NCH_2_), 1.77 (m, 2H, CH_2_), 1.42 (m, 4H, 2xCH_2_), 0.90 (t, *J* = 7.3 Hz, 3H, CH_3_). *δ*_C_ (100 MHz; DMSO-*d_6_*) 176.3 (C=O), 144.0 (Ar-C), 141.2 (Ar-C), 134.0 (Ar-C), 133.8 (Ar-C), 127.3 (Ar-C), 124.0 (Ar-C), 123.5 (Ar-C), 121.0 (Ar-C), 120.4 (Ar-C), 117.2 (Ar-C), 115.8 (Ar-C), 107.7 (Ar-C), 45.4
(N-CH_2_), 28.8 (CH_2_), 27.3 (CH_2_), 22.5 (CH_2_), 14.5 (CH_2_). HRMS (+NSI) Found 281.1650 (Calcd. 281.1648 for C_18_H_21_N_2_O; [M+H]^+^). M.Pt. 199.1-199.9 ºC. ν_max_/cm^-1^ 3416w, 3325w, 2922w, 263w, 1584s, 1494s, 1463m, 1227m, 1174m, 805m, 746s, 685m. λ_EX_ = 435 nm, λ_EM_ = 545 nm (EtOH).

### 2-Amino-10-benzylacridone, 5c

Compound **5c** was prepared from 10-benzyl-2-nitroacridone (1.72 g, 5.21 mmol) and obtained as a red crystalline powder (1.52 g, 97%) which was used without further purification. *δ*_H_ (270 MHz; DMSO-*d_6_*) 8.35 (dd, *J* = 7.9 Hz,
*J* = 1.8 Hz, 1H, H8), 7.66 (ddd, *J* = 8.7 Hz, *J* = 6.7 Hz, *J* = 1.8 Hz, 1H, H6), 7.57-7.53 (m, 2H, 2xAr-H), 7.45 (d,
*J* = 9.2 Hz, 1H, H4), 7.36-7.21 (m, 4H, 4xAr-H), 7.14-7.09 (m, 3H, 3xAr-H), 5.75 (s, 2H, NH_2_), 5.31 (s, 2H, CH_2_). *δ*_C_ (101 MHz; DMSO-*d_6_*) 176.6 (C=O), 144.3 (Ar-C), 141.9 (Ar-C), 137.3 (Ar-C), 134.5 (Ar-C), 133.9 (Ar-C), 129.3 (Ar-C), 127.7 (Ar-C), 127.2 (Ar-C), 126.3 (Ar-C), 123.9 (Ar-C), 123.6 (Ar-C), 121.1 (Ar-C), 120.8 (Ar-C), 117.6
(Ar-C), 116.2 (Ar-C), 107.7 (Ar-C), 49.2 (CH_2_). HRMS (+NSI) Found 301.1339 (Calcd. 301.1135 for C_20_H_17_N_2_O; [M+H]^+^). M.Pt. 201.4-202.5 ºC. ν_max­_/cm^-1^ 3420w, 3331w, 3329w, 2357w, 1615m, 1587s, 1574s, 1494s, 1464s, 1278m, 1174m, 809m, 751s, 727s, 692m. λ_EX_ = 429 nm, λ_EM_ = 550 nm (EtOH).

## General procedure for the preparation of Boc-protected amino acid derivatives of *N*-substituted-2-aminoacridones.

The Boc-protected amino acid derivative, R-OH (1.05 eq.) was added to dry THF and the mixture cooled to
-12 ºC in a salt ice bath with stirring. To this was added *N*-methyl morpholine (NMM; 1.00 eq.) and isobutyl chloroformate (^i^BuCF; 1.00 eq.) followed 90 s later by a previously cooled solution of amines **5a-c** (1.00 eq.) in dry THF. The mixture was stirred overnight. The solvent was evaporated and replaced by DCM. The mixture was washed sequentially with aqueous citric acid (0.1 M), aqueous saturated NaHCO_3_ solution, water and then brine. The organic layer was dried over MgSO_4_ and solvent evaporated to yield the crude product.

### 2-(Boc-L-alanylamino)-10-methylacridone, 6a

Compound **6a** was prepared from amine **5a** (182 mg, 0.82 mmol) and Boc-L-alanine (161 mg, 0.88 mmol) and the crude product recrystallised from ^i^PrOH/^i^PrO_2_ as an orange crystalline powder (66 mg, 20%). *δ*_H_ (400 MHz; CDCl_3_) 8.53 (dd, *J* = 7.8 Hz, *J* = 1.4 Hz, 1H, Ar-H), 8.29 (d, *J* = 1.4 Hz, 1H, Ar-H), 7.69 (t, *J* = 7.3 Hz, 1H, Ar-H), 7.50 (d, *J* = 10.1 Hz, 1H, Ar-H), 7.45 (d, *J* = 8.7 Hz, 1H, Ar-H), 7.29-7.21 (m, 2 x Ar-H), 3.86 (s, 3H, NCH_3_), 1.59 (broad s, 1H, CHCH_3_), 1.48 (s, 9H, C(CH_3_)_3_), 1.46 (s, 3H, CHCH_3_). *δ*_C_ (101 MHz; CDCl_3_) 176.7 (C=O), 172.4 (C=O), 155.8 (C=O), 142.5 (Ar-C), 139.0 (Ar-C), 134.4 (Ar-C), 133.6 (Ar-C), 127.1 (Ar-C), 126.8 (Ar-C), 122.2 (Ar-C), 121.6 (Ar-C), 121.5 (Ar-C), 117.3 (Ar-C), 116.6 (Ar-C), 115.8 (Ar-C), 78.6 (CHCH_3_), 51.0 (C(CH_3_)_3_), 34.1 (NCH_3_) 28.8 (C(CH_3_)_3_), 18.6 (CHCH_3_). HRMS (+NSI) Found 396.1920 (Calcd. 396.1918 for C_22_H_26_N_3_O_4_; [M+H]^+^). M.Pt. 204.5-205.6 ºC. ν_max­_/cm^-1^ 3399w, 3286w, 2979w, 1715m, 1683s, 1594s, 1526s, 1485s, 1159s (broad), 1047s, 1026s, 763s, 740s.

### 2-(Boc-L-alanylamino)-10-pentylacridone, 6b

Compound **6b** was prepared from amine **5b** (95 mg, 0.34 mmol) and Boc-L-alanine (68 mg, 0.36 mmol) and the crude product (138 mg, 91%) suitable for use without further purification. *δ*_H_ (400 MHz; DMSO-*d_6_*) 10.21 (s, NH), 8.62 (d, *J* = 2.8 Hz, 1H, Ar-H), 8.35 (d, *J* = 8.1 Hz, 1H, Ar-H), 8.07 (dd, *J* = 9.2 Hz, *J* = 2.8 Hz, 1H, Ar-H), 7.85-7.79 (m, 3H, 2 x Ar-H and NH), 7.32 (ddd, *J* = 7.8 Hz, *J* = 6.0 Hz, *J* = 1.8 Hz, 1H, Ar-H), 7.15 (d, *J* = 7.3 Hz, 1H, Ar-H), 4.47 (t, *J* = 7.3 Hz, 2H, NCH_2_), 4.18-4.11 (m, 1H, CHCH_3_), 1.79 (m, 2H, CH_2_), 1.53-1.45 (m, 2H, CH_2_), 1.46-1.36 (m, 11H, CCH_3_ and CH_2_), 1.30 (d, *J* = 7.3 Hz, 3H, CHCH_3_), 0.91 (t, *J* = 7.3 Hz, 3H, CH_3_). *δ*_C_ (100 MHz; DMSO-*d_6_*) 176.6 (C=O), 172.4 (C=O), 155.7 (C=O), 141.7 (Ar-C), 138.2 (Ar-C), 134.6 (Ar-C), 133.7 (Ar-C), 127.3 (Ar-C), 127.1
(Ar-C), 122.2 (Ar-C), 121.6 (Ar-C), 121.5 (Ar-C), 117.1 (Ar-C), 116.3 (Ar-C), 116.0 (Ar-C), 78.6 (C(CH_3_)_3_), 51.0 (CHCH_3_), 45.7 (CH_2_), 28.8 (C(CH_3_)_3_), 28.7 (CH_2_), 27.2 (CH_2_), 22.6 (CH_2_), 18.5 (CHCH_3_), 14.5 (CH_2_CH_3_). HRMS (+NSI) Found 452.2541 (Calcd. 452.2544 for C_26_H_34_N_3_O_4_; [M+H]^+^). M.Pt. 167.0-167.9 ºC. ν_max_/cm^-1^ 3298w (broad), 2928w, 1686m, 1595s, 1568s, 1538s, 1493s, 1465s, 1269m, 1164 s(broad), 751s, 687m.

### 2-(Boc-L-alanylamino)-10-benzylacridone, 6c

Compound **6c** was obtained from amine **5c** (600 mg, 2.00 mmol) and Boc-L-alanine (397 mg, 2.10 mmol) and the crude product recrystallised from aq. EtOH as an orange crystalline powder (674 mg, 72%). *δ*_H_ (400 MHz; DMSO-*d_6_*) 10.17 (s, 1H, NH), 8.63 (d, *J* = 2.6 Hz, 1H, H1), 8.34 (dd, *J* = 7.8 Hz, *J* = 1.8 Hz, 1H, H8), 7.92 (dd, *J* = 9.2 Hz,
*J* = 2.6 Hz, 1H, H3), 7.69 (ddd, *J* = 8.7 Hz, *J* = 6.9 Hz,*J* = 1.8 Hz, 1H, H6), 7.63 (d, *J* = 9.2 Hz, 1H, H4), 7.58 (d,
*J* = 8.7 Hz, 1H, H5), 7.30-7.25 (m, 3H, 3xAr-H), 7.23-7.20 (m, 1H, Ar-H), 7.11-7.09 (m, 2H, 2xAr-H), 5.76 (s, 2H, CH_2_), 4.13-4.06 (m, 1H, CHCH_3_), 1.34 (s, 9H, C(CH_3_)_3_), 1.25 (d, *J* = 7.3 Hz, 3H, CHCH_3_). *δ*_C_ (101 MHz; DMSO-*d_6_*) 176.9 (C=O), 172.5 (C=O), 155.8 (C=O), 142.3 (Ar-C), 138.8 (Ar-C), 136.9 (Ar-C), 134.6 (Ar-C), 133.9 (Ar-C), 129.4 (Ar-C), 127.8 (Ar-C), 127.3 (Ar-C), 127.1 (Ar-C), 126.3 (Ar-C), 122.3 (Ar-C), 121.8 (Ar-C), 121.7 (Ar-C), 117.3 (Ar-C), 116.6 (Ar-C), 116.1 (Ar-C), 78.6 (C(CH_3_)_3_), 51.0 (CH_2_), 49.4 (CHCH_3_), 28.7 (C(CH_3_)_3_), 18.5 (CHCH_3_). HRMS (+NSI) Found 472.2228 (Calcd. 472.2231 for C_28_H_30_N_3_O_4_; [M+H]^+^). M.Pt. 211.3-211.8 ºC. ν_max­_/cm^-1^ 3188m (broad), 2976w, 1690s, 1596s, 1567s, 1535s, 1505s, 1274s, 1168s, 749s, 688s.

### 2-(Boc-L-alanyl-L-alanylamino)-10-benzylacridone, 8

Compound **8** was obtained from amine **5c** (300 mg, 1.00 mmol) and Boc-L-alanyl-L-alanine (273 mg, 1.05 mmol) and the crude product recrystallised from ^i^PrOH/^i^PrO_2_ as a beige crystalline powder (324 mg, 60%). *δ*_H_ (400 MHz; DMSO-*d_6_*) 10.18 (s, 1H, NH), 8.62 (d, *J* = 2.8 Hz, 1H, Ar-H), 8.32 (dd, *J* = 7.8 Hz, *J* = 1.8 Hz, 1H, Ar-H), 8.03 (d, *J* = 7.3 Hz, 1H, NH), 7.91 (dd, *J* = 9.2 Hz, *J* = 2.8 Hz, 1H, Ar-H), 7.70 (ddd, *J* = 8.7 Hz, *J* = 6.9 Hz, *J* = 1.4 Hz, 1H, Ar-H), 7.63 (d, *J* = 9.6 Hz, 1H, Ar-H), 7.59 (d, *J* = 9.2 Hz, 1H, Ar-H), 7.30-7.20 (m, 4H, 4xAr-H), 7.10 (d,
*J* = 6.9 Hz, 2H, 2xAr-H), 6.96 (d, *J* = 7.3 Hz, 1H, NH), 5.77 (s, 2H, CH_2_), 4.37 (quint, *J* = 6.9 Hz, 1H, CHCH_3_), 3.97 (quint, *J* = 7.3 Hz, 1H, CHCH_3_), 1.33 (s, 9H, C(CH_3_)_3_), 1.30 (d, *J* = 7.3 Hz, 3H, CHCH_3_), 1.16 (d, *J* = 7.3 Hz, 3H, CHCH_3_). *δ*_C_ (101 MHz; DMSO-*d_6_*) 176.9 (C=O), 173.1 (C=O), 171.7 (C=O), 155.7 (C=O), 142.3 (Ar-C), 138.9 (Ar-C), 136.9 (Ar-C), 134.7 (Ar-C), 133.8 (Ar-C), 129.4 (Ar-C), 127.8 (Ar-C), 127.3 (Ar-C), 127.1 (Ar-C), 126.3 (Ar-C), 122.3 A(r-C), 121.9 (Ar-C), 121.7 (Ar-C), 117.4 (Ar-C), 116.6 (Ar-C), 116.1 (Ar-C), 78.6 (C(CH_3_)_3_), 50.1(CH_2_), 49.5 (CHCH_3_), 49.4 (CHCH_3_), 28.7 (C(CH_3_)_3_), 18.7 (CHCH_3_), 18.6 (CHCH_3_). HRMS (+NSI) Found 543.2598 (Calcd. 543.2602 for C_31_H_35_N_4_O_5_; [M+H]^+^). M.Pt. 188.5-190.1 ºC. ν_max­_/cm^-1^ 3258m, 2979w, 1714m, 1634s, 1614s, 1596s, 1488s, 1364m, 1269m, 1178s (broad), 750s, 699s.

### 2-(Boc-β-alanylamino)-10-benzylacridone, 10

Compound **10** was obtained from amine **5c** (300 mg, 1.00 mmol) and Boc-β-alanine (199 mg, 1.05 mmol) and the crude product recrystallised from ^i^PrOH/^i^PrO_2_ as an orange crystalline powder (227 mg, 48%). *δ*_H_ (400 MHz; DMSO-*d_6_*) 10.15 (s, 1H, NH), 8.61 (d, *J* = 2.3 Hz, 1H, Ar-H), 8.33 (dd, *J* = 8.2 Hz, *J* = 1.4 Hz, 1H, Ar-H), 7.90 (dd,
*J* = 9.2 Hz, *J* = 2.3 Hz, 1H, Ar-H), 7.69 (ddd, *J* = 8.7 Hz, *J* = 6.9 Hz, *J* = 1.8 Hz, 1H, Ar-H), 7.61 (d, *J* = 9.6 Hz, 1H, Ar-H), 7.58 (d, *J* = 8.7 Hz, 1H, Ar-H), 7.30-7.20 (m, 4H, 4xAr-H), 7.10 (d, *J* = 7.3 Hz, 2H, 2xAr-H), 6.88 (t,
*J* = 5.5 Hz, 1H, NH), 5.76 (s, 2H, Ar-CH_2_), 3.21 (q, *J* = 6.4 Hz, 2H, CH_2_), 2.48-2.44 (m, CH_2_), 1.33 (s, 9H, C(CH_3_)_3_). *δ*_C_ (101 MHz; DMSO-*d_6_*) 176.9 (C=O), 169.9 (C=O), 156.1 (C=O), 142.3 (Ar-C), 138.7 (Ar-C), 136.9 (Ar-C), 134.7 (Ar-C), 134.1 (Ar-C), 129.4 (Ar-C), 127.8 (Ar-C), 127.3 (Ar-C), 127.1 (Ar-C), 126.4 (Ar-C), 122.3 (Ar-C), 121.8
(Ar-C), 121.7 (Ar-C), 117.3 (Ar-C), 116.6 (Ar-C), 115.9 (Ar-C), 78.2 (C(CH_3_)_3_), 49.4 (Ar-CH_2_), 37.3 (CH_2_), 37.1 (CH_2_), 28.8 (C(CH_3_)_3_). HRMS (+NSI) Found 472.2228 (Calcd. 472.2231 for C_28_H_30_N_3_O_4_; [M+H]^+^). M.Pt.
121.1-123.3 ºC. ν_max­_/cm^-1^ 3349w (broad), 1678m, 1581s, 1504s, 1487s, 1275s, 1164s (broad), 757s.

## General procedure for the deprotection of Boc-protected amino acid derivatives of *N*-substituted 2-aminoacridones.

The Boc-protected substrate was added to TFA (5 mL) with stirring at room temperature. After 2 h excess TFA was evaporated and the residue triturated with Et_2_O (5 mL). The resulting precipitate was filtered by vacuum to yield the product.

### 2-(L-Alanylamino)-10-methylacridone TFA salt, 7a

Compound **7a** was obtained from compound **6a** (37 mg, 0.09 mmol) as a yellow-green powder (35 mg, 91%). *δ*_H_ (400 MHz; DMSO-*d_6_*) 10.72 (s, 1H, NH), 8.59 (d, *J* = 2.8 Hz, 1H, Ar-H), 8.29 (d, *J* = 8.2 Hz, 1H, Ar-H), 8.23 (broad s, 3H, NH_3_), 8.04 (dd, *J* = 9.2 Hz, *J* = 2.3 Hz, 1H, Ar-H), 7.88 (d, *J* = 9.2 Hz, 1H, Ar-H), 7.84-7.77 (m, 2H, 2xAr-H), 7.30 (t, *J* = 6.4 Hz, 1H, Ar-H), 4.02 (q, *J* = 6.9 Hz, 1H, CHCH_3_), 3.91 (s, 3H, NCH_3_), 1.46 (d, *J* = 6.9 Hz, 3H, CHCH_3_). *δ*_C_ (101 MHz; DMSO-*d_6_*) 176.7 (C=O), 168.7 (C=O), 142.6 (Ar-C), 139.5 (Ar-C), 134.6 (Ar-C), 132.6 (Ar-C), 127.0 (Ar-C), 126.8 (Ar-C), 122.2 (Ar-C), 121.7 (Ar-C), 121.7 (Ar-C), 117.7 (Ar-C), 116.7 (Ar-C), 116.3
(Ar-C), 49.6 (CHCH_3_), 34.3 (NCH_3_), 17.7 (CHCH_3_). HRMS (+NSI) Found 296.1391 (Calcd. 296.1394 for C_17_H_18_N_3_O_2_; [M+H]^+^). M.Pt. Decomp. >110 ºC. ν_max­_/cm^-1^ 3200-2700w (broad), 1671s, 1580s, 1504s, 1465s, 1180s, 1129s, 753m, 721s. λ_EX_ = 418 nm, λ_EM_ = 453 nm (EtOH). Copies of the NMR spectra for this compound are provided as **appendices S1** and **S2**.

### 2-(L-Alanylamino)-10-pentylacridone TFA salt, 7b

Compound **7b** was obtained from compound **6b** (104 mg, 0.23 mmol) as a dark yellow powder (99 mg, 93%). *δ*_H_ (400 MHz; DMSO-*d_6_*) 10.76 (s, 1H, NH), 8.66 (d, *J* = 2.8 Hz, 1H, Ar-H), 8.36 (d, *J* = 7.8 Hz, 1H, Ar-H), 8.32 (s [broad], 3H, NH_3_), 8.06 (dd, *J* = 9.6, *J* = 2.8 Hz, 1H, Ar-H), 7.90 (d, *J* = 9.6 Hz, 1H, Ar-H), 7.85-7.81 (m, 2H, 2 x
Ar-H), 7.34 (ddd, *J* = 7.8 Hz, *J* = 6.0 Hz, *J* = 1.8 Hz, 1H, Ar-H), 4.48 (t, *J* = 7.6 Hz, 2H, NCH_2_), 4.11-4.05 (m, 1H, CHCH_3_), 1.80 (pent, *J* = 7.6 Hz, 2H, CH_2_), 1.52-1.46 (m, 5H, CH_2_ and CHCH_3_), 1.44-1.35 (m, 2H, CH_2_), 0.91 (t,
*J* = 7.6 Hz, 3H, CH_2_CH_3_). *δ*_C_ (100 MHz; DMSO-*d_6_*) 176.0 (C=O), 168.1 (C=O), 141.1 (Ar-C), 138.0 (Ar-C), 134.1 (Ar-C), 132.0 (Ar-C) 126.7 (Ar-C), 126.5 (Ar-C), 121.6 (Ar-C), 121.1 (Ar-C), 121.1 (Ar-C), 116.9 (Ar-C), 115.9
(Ar-C), 115.8 (Ar-C), 49.0 (CHCH_3_), 45.1 (NCH_2_), 28.1 (CH_2_), 26.6 (CH_2_), 21.9 (CH_2_), 17.1 (CHCH_3_), 13.9 (CH_2_CH_3_). HRMS (+NSI) Found 352.2019 (Calcd. 352.2020 for C_21_H_26_N_3_O_2_; [M+H]^+^). M.Pt. Decomp. >90 ºC. ν_max_/cm^-1^ 2936w (broad),1670m, 1579 m, 1545m, 1502s, 1465m, 1178s, 1137s, 798m, 754s, 722s. λ_EX_ = 401/418 nm, λ_EM_ = 445 nm (EtOH). Copies of the NMR spectra for this compound are provided as **appendices S3** and **S4**.

### 2-(L-Alanylamino)-10-benzylacridone TFA salt, 7c

Compound **7c** was obtained from compound **6c** (37 mg, 0.08 mmol) as a yellow powder (35 mg, 92%). *δ*_H_ (400 MHz; DMSO-*d_6_*) 10.69 (s, 1H, NH), 8.68 (d, *J* = 2.8 Hz, 1H, Ar-H), 8.38 (dd, *J* = 7.8 Hz, *J* = 1.8 Hz, 1H, Ar-H), 8.25 (broad s, 3H, NH_3_), 7.94 (dd, *J* = 9.4 Hz, *J* = 2.8 Hz, 1H, Ar-H), 7.76 (ddd, *J* = 8.7 Hz, *J* = 6.9 Hz, *J* = 1.8 Hz, 1H, Ar-H), 7.72 (d, *J* = 9.4 Hz, 1H, Ar-H), 7.65 (d, *J* = 8.7 Hz, 1H, Ar-H), 7.37-7.26 (m, 4H, 4xAr-H), 7.16 (d,
*J* = 7.3 Hz, 2H, 2xAr-H), 5.83 (s, 2H, CH_2_), 4.01 (broad s, 1H, CH), 1.49 (d, *J* = 6.9 Hz, 3H, CH_3_). *δ*_C_ (101 MHz; DMSO-*d_6_*) 176.9 (C=O), 168.7 (C=O), 142.4 (Ar-C), 139.3 (Ar-C), 136.8 (Ar-C), 134.8 (Ar-C), 132.9 (Ar-C), 129.4 (Ar-C), 127.9 (Ar-C), 127.2 (Ar-C), 126.3 (Ar-C), 122.3 (Ar-C), 122.1 (Ar-C), 121.8 (Ar-C), 117.8 (Ar-C), 116.7
(Ar-C), 116.5 (Ar-C), 49.6 (CH_2_), 49.4 (CH), 17.7 (CH_3_). HRMS (+NSI) Found 372.1706 (Calcd. 372.1707 for C_23_H_22_N_3_O_2_; [M+H]^+^). ν_max­_/cm^-1^ 3200-2300w (broad), 1672s, 1581s, 1503s, 1466m, 1180s (broad), 1132s (broad), 722s. λ_EX_ = 415 nm, λ_EM_ = 445 nm (EtOH). Copies of the NMR spectra for this compound are provided as **appendices S5** and **S6**.

### 2-(L-Alanyl-L-alanylamino)-10-benzylacridone TFA salt, 9

Compound **9** was obtained from compound **8** (103 mg, 0.19 mmol) as a yellow-green crystalline powder
(104 mg, 98%). *δ*_H_ (400 MHz; DMSO-*d_6_*) 10.40 (s, 1H, NH), 8.75 (d, *J* = 6.9 Hz, 1H, Ar-H), 8.68 (d, *J* = 2.8 Hz, 1H, Ar-H), 8.38 (dd, *J* = 7.8 Hz,*J* = 1.8 Hz, 1H, Ar-H), 8.10 (broad s, 3H, NH_3_), 7.93 (dd, *J* = 9.2 Hz, *J* = 2.8 Hz, 1H,
Ar-H), 7.75 (ddd, *J* = 8.7 Hz, *J* = 6.9 Hz, *J* = 1.4 Hz, 1H, Ar-H), 7.69 (d, *J* = 9.6 Hz, 1H, Ar-H), 7.64 (d, *J* = 9.2 Hz, 1H, Ar-H), 7.35-7.25 (m, 4H, 4xAr-H), 7.15 (d, *J* = 6.9 Hz, 2H, 2xAr-H), 5.82 (s, 2H,CH_2_), 4.64-4.54 (m, 1H, CHCH_3_), 3.93-3.87 (m, 1H, CHCH_3_), 1.39 (d, *J* = 6.9 Hz, 6H, 2xCHCH_3_). *δ*_C_ (101 MHz; DMSO-*d_6_*) 176.4 (C=O), 170.7 (C=O), 169.3 (C=O), 158.1([F_3_CCO_2_]^-^), 157.8 (Ar-C), 141.8 (Ar-C), 138.4 (Ar-C), 136.4 (Ar-C), 134.3 (Ar-C), 133.2 (Ar-C), 128.9 (Ar-C), 127.3 (Ar-C), 126.7 (Ar-C), 126.6 (Ar-C), 125.8 (Ar-C), 121.8 (Ar-C), 121.4 (Ar-C), 121.2 (Ar-C), 117.0 (Ar-C), 116.1 ([F_3_CCO_2_]^-^), 115.6 (Ar-C), 49.3 (CH_2_), 48.9 (CH), 48.1 (CH), 18.1 (CH_3_), 17.2 (CH_3_). HRMS (+NSI) Found 443.2076 (Calcd. 443.2078 for C_26_H_27_N_4_O_3_; [M+H]^+^). ν_max_/cm^-1^ 3300-2600w (broad), 1667s, 1597m, 1504s, 1178s, 1131s, 722s. λ_EX_ = 414 nm, λ_EM_ = 450 nm (EtOH). Copies of the NMR spectra for this compound are provided as **appendices S7** and **S8.**

### 2-(β-Alanylamino)-10-benzylacridone, 11

Compound **11** was obtained from compound **10** (109 mg, 0.23 mmol) as a tan powder. *δ*_H_ (400 MHz; DMSO-*d_6_*) 10.41 (s, 1H, NH), 8.70 (d, *J* = 2.8 Hz, 1H, Ar-H), 8.37 (dd, *J* = 8.2 Hz, *J* = 1.4 Hz, 1H, Ar-H), 7.92 (dd,
*J* = 9.6 Hz, *J* = 2.8 Hz, 1H, Ar-H), 7.77 (broad s, 3H, NH_3_), 7.75 (ddd, *J* = 8.7 Hz, *J* = 7.3 Hz, *J* = 1.4 Hz, 1H, Ar-H), 7.68 (d, *J* = 9.2 Hz, 1H, Ar-H), 7.64 (d, *J* = 8.7 Hz, 1H, Ar-H), 7.35-7.25 (m, 4H, 4xAr-H), 7.15 (d, *J* = 7.3 Hz, 2H, 2xAr-H), 5.82 (s, 2H, Ar-CH_2_), 3.12 (q, *J* = 6.0 Hz, 2H, CH_2_), 2.74 (t, *J* = 6.4 Hz, 2H, CH_2_). *δ*_C_ (101 MHz; DMSO-*d_6_*) 176.9 (C=O), 168.9 (C=O), 142.3 (Ar-C), 138.9 (Ar-C), 136.9 (Ar-C), 134.7 (Ar-C), 133.7 (Ar-C0, 129.4 (Ar-C), 127.8 (Ar-C), 127.2 (Ar-C), 127.1 (Ar-C), 126.8 (Ar-C), 122.3 (Ar-C), 121.9 (Ar-C), 121.7 (Ar-C), 117.4 (Ar-C), 116.6 (Ar-C), 116.1 (Ar-C), 49.4 (Ar-CH_2_), 35.5 (CH_2_), 33.7 (CH_2_). HRMS (+NSI) Found 372.1712 (Calcd. 372.1707 for C_23_H_22_N_3_O_2_; [M+H]^+^). ν_max­_/cm^-1^ 3200-2600w (broad), 1668s, 1575s, 1504s, 1465s, 1177s, 1128s, 755s, 721s. λ_EX_ = 414 nm, λ_EM_ = 449 nm (EtOH). Copies of the NMR spectra for this compound are provided as **appendices S9** and **S10**.

## Microbiological screening procedure.

Substrates **7a-c**, **9** and **11** (5 or 10 mg as stated) were dissolved in *N*-methyl pyrrolidone (200 μL) and added to sterile molten Columbia agar [powder base (4 g) in deionised water (100 mL)] at 50 ºC to a final concentration of
50/100 mg L^-1^. The mixtures were each divided among five sterile Petri dishes and allowed to set before inoculating one plate with suspensions of twenty microorganisms (as listed in tables S1-5) in deionised water (at an approximate concentration of 100 000 cfu/spot measured as 0.5 MacFarland units by turbidity). The remaining plates were inoculated with suspensions of microorganisms selected for their expected positive/negative activity using the streak plate method to obtain individual colony growth.

Incubation of the plates was at 37 ºC for 18 h before analysis. Illumination of the plates was *via* a UVP UVLS-58 ultraviolet light box at a wavelength of 360 nm. Photographs were obtained using an Olympus FE-240 with the flash disabled in the automatic exposure mode. Photographs are for illustrative purposes only as colours shown may not be as seen by eye. Results tables include subjective assessment of colony growth, colour and fluorescence in comparison to the control plates.

## 3. Full tables of microorganism screening results.

**S1 Table. Full screening results for substrates 7a-c at 50 mg L^-1^.**

|  |  |  | **Substrate** | | | | | |
| --- | --- | --- | --- | --- | --- | --- | --- | --- |
|  |  | **Control** | **7a** | | **7b** | | **7c** | |
|  | **Microorganism / Reference^a^** | **Growth^b^** | **Growth^b^** | **Fluorescence^c^** | **Growth^b^** | **Fluorescence^c^** | **Growth^b^** | **Fluorescence^c^** |
|  | Gram-negative microorganisms |  |  |  |  |  |  |  |
| 1 | *Escherichia coli* NCTC 10418 | ++ | - | - | - | - | ++ | ++ yellow |
| 2 | *Klebsiella pneumoniae* NCTC 9528 | ++ | - | - | - | - | ++ | - |
| 3 | *Providencia rettgeri* NCTC 7475 | ++ | ++ | ++ yellow | ++ | + yellow | ++ | ++ yellow |
| 4 | *Enterobacter cloacae* NCTC 11936 | ++ | ++ | ++ yellow | ++ | + yellow | ++ | ++ yellow |
| 5 | *Serratia marcescens* NCTC 10211 | ++ | ++ | ++ yellow | ++ | ++ yellow | ++ | ++ yellow |
| 6 | *Salmonella typhimurium* NCTC 74 | ++ | ++ | ++ yellow | ++ | + yellow | ++ | +/- yellow |
| 7 | *Pseudomonas aeruginosa* NCTC 10662 | ++ | ++ | ++ yellow | ++ | + yellow | ++ | ++ yellow |
| 8 | *Yersinia enterocolitica* NCTC 11176 | ++ | ++ | + yellow | ++ | + yellow | ++ | + yellow |
| 9 | *Burkholderia cepacia* NCTC 10743 | ++ | ++ | ++ yellow | ++ | + blue | ++ | ++ blue |
| 10 | *Acinetobacter baumannii* NCTC 12156 | ++ | ++ | ++ yellow | - | - | + | + yellow |
|  | Gram-positive microorganisms |  |  |  |  |  |  |  |
| 11 | *Streptococcus pyogenes* NCTC 8306 | + | - | - | - | - | - | - |
| 12 | *Staphylococcus aureus* (MRSA) NCTC 11939 | **+** | - | - | - | - | + | + blue |
| 13 | *Staphylococcus aureus*  (MSSA) NCTC 6571 | **+** | - | - | - | - | + | + blue |
| 14 | *Staphylococcus epidermidis* NCTC 11047 | **+** | - | - | - | - | - | - |
| 15 | *Listeria monocytogenes* NCTC 11994 | **+** | + | - | Tr. | - | +/- | - |
| 16 | *Enterococcus faecium* NCTC 7171 | **+** | + | - | + | Tr. yellow | + | Tr. yellow |
| 17 | *Enterococcus faecalis* NCTC 775 | **+** | + | - | + | +/- yellow | + | Tr. yellow |
| 18 | *Bacillus subtilis* NCTC 9372 | + | + | - | - | - | - | - |
|  | Yeasts |  |  |  |  |  |  |  |
| 19 | *Candida albicans* ATCC 90028 | + | + | - | + | +/- blue | + | - |
| 20 | *Candida glabrata* NCPF 3943 | + | Tr. | - | Tr. | - | + | - |
|  | Background |  |  | + blue |  | + blue |  | + blue |

^a^ NCTC: National Collection of Type Cultures; ATCC: American Type Culture Collection; NCPF: National Collection of Pathogenic Fungi.

^b^ ++ strong growth, + moderate growth, +/- weak growth, Tr. trace of growth, - no growth.

^c^ ++ strong fluorescence, + moderate fluorescence, +/- weak fluorescence, Tr. trace of fluorescence, - no fluorescence.

**S2 Table. Full screening results for substrate 7c at 100 mg L^-1^ (with 50 mg L^-1^ shown for comparison).**

|  |  | **Substrate / Concentration** | | | |
| --- | --- | --- | --- | --- | --- |
|  |  | **7c**  **50 mg L^-1^** | | **7c**  **100 mg L^-1^** | |
|  | **Microorganism / Reference^a^** | **Growth^b^** | **Fluorescence^c^** | **Growth^b^** | **Fluorescence^c^** |
|  | Gram-negative microorganisms |  |  |  |  |
| 1 | *Escherichia coli* NCTC 10418 | ++ | ++ yellow | - | - |
| 2 | *Klebsiella pneumoniae* NCTC 9528 | ++ | - | - | - |
| 3 | *Providencia rettgeri* NCTC 7475 | ++ | ++ yellow | + | +/- yellow |
| 4 | *Enterobacter cloacae* NCTC 11936 | ++ | ++ yellow | + | +/- yellow |
| 5 | *Serratia marcescens* NCTC 10211 | ++ | ++ yellow | + | +/- yellow |
| 6 | *Salmonella typhimurium* NCTC 74 | ++ | +/- yellow | + | +/- yellow |
| 7 | *Pseudomonas aeruginosa* NCTC 10662 | ++ | ++ yellow | + | - |
| 8 | *Yersinia enterocolitica* NCTC 11176 | ++ | + yellow | + | +/- yellow |
| 9 | *Burkholderia cepacia* NCTC 10743 | ++ | ++ blue | + | +/- yellow |
| 10 | *Acinetobacter baumannii* NCTC 12156 | + | + yellow | - | - |
|  | Gram-positive microorganisms |  |  |  |  |
| 11 | *Streptococcus pyogenes* NCTC 8306 | - | - | - | - |
| 12 | *Staphylococcus aureus* (MRSA) NCTC 11939 | + | + blue | - | - |
| 13 | *Staphylococcus aureus* (MSSA) NCTC 6571 | + | + blue | - | - |
| 14 | *Staphylococcus epidermidis* NCTC 11047 | - | - | - | - |
| 15 | *Listeria monocytogenes* NCTC 11994 | +/- | - | +/- | - |
| 16 | *Enterococcus faecium* NCTC 7171 | + | Tr. yellow | +/- | - |
| 17 | *Enterococcus faecalis* NCTC 775 | + | Tr. yellow | +/- | - |
| 18 | *Bacillus subtilis* NCTC 9372 | - | - | - | - |
|  | Yeasts |  |  |  |  |
| 19 | *Candida albicans* ATCC 90028 | + | - | +/- | - |
| 20 | *Candida glabrata* NCPF 3943 | + | - | +/- | - |
|  | Background |  | + blue |  | + blue |

^a^ NCTC: National Collection of Type Cultures; ATCC: American Type Culture Collection; NCPF: National Collection of Pathogenic Fungi.

^b^ ++ strong growth, + moderate growth, +/- weak growth, Tr. trace of growth, - no growth.

^c^ ++ strong fluorescence, + moderate fluorescence, +/- weak fluorescence, Tr. trace of fluorescence, - no fluorescence.

**S3 Table. Full screening results for substrates 9 and 11 at 50 mg L^-1^.**

|  |  | **Substrate** | | | |
| --- | --- | --- | --- | --- | --- |
|  |  | **9** | | **11** | |
|  | **Microorganism / Reference^a^** | **Growth^b^** | **Fluorescence^c^** | **Growth^b^** | **Fluorescence^c^** |
|  | Gram-negative microorganisms |  |  |  |  |
| 1 | *Escherichia coli* NCTC 10418 | ++ | + yellow | + | +/- blue |
| 2 | *Klebsiella pneumoniae* NCTC 9528 | ++ | - | + | +/- blue |
| 3 | *Providencia rettgeri* NCTC 7475 | ++ | ++ yellow | ++ | +/- blue |
| 4 | *Enterobacter cloacae* NCTC 11936 | ++ | + yellow | + | +/- blue |
| 5 | *Serratia marcescens* NCTC 10211 | ++ | ++ yellow | ++ | ++ yellow |
| 6 | *Salmonella typhimurium* NCTC 74 | ++ | Tr. yellow | ++ | +/- blue |
| 7 | *Pseudomonas aeruginosa* NCTC 10662 | ++ | + yellow | ++ | ++ yellow |
| 8 | *Yersinia enterocolitica* NCTC 11176 | ++ | +/- yellow | ++ | +/- blue |
| 9 | *Burkholderia cepacia* NCTC 10743 | ++ | - | ++ | +/- blue |
| 10 | *Acinetobacter baumannii* NCTC 12156 | ++ | ++ yellow | ++ | +/- blue |
|  | Gram-positive microorganisms |  |  |  |  |
| 11 | *Streptococcus pyogenes* NCTC 8306 | + | **-** | - | **-** |
| 12 | *Staphylococcus aureus* (MRSA) NCTC 11939 | + | Tr. blue | + | +/- blue |
| 13 | *Staphylococcus aureus*  (MSSA) NCTC 6571 | + | Tr. blue | + | Tr. blue |
| 14 | *Staphylococcus epidermidis* NCTC 11047 | + | - | - | - |
| 15 | *Listeria monocytogenes* NCTC 11994 | + | **-** | + | Tr. blue |
| 16 | *Enterococcus faecium* NCTC 7171 | + | **-** | + | Tr. blue |
| 17 | *Enterococcus faecalis* NCTC 775 | + | **-** | + | Tr. blue |
| 18 | *Bacillus subtilis* NCTC 9372 | + | - | - | **-** |
|  | Yeasts |  |  |  |  |
| 19 | *Candida albicans* ATCC 90028 | + | **-** | + | Tr. blue |
| 20 | *Candida glabrata* NCPF 3943 | + | **-** | + | **-** |
|  | Background |  | + blue |  | + blue |

^a^ NCTC: National Collection of Type Cultures; ATCC: American Type Culture Collection; NCPF: National Collection of Pathogenic Fungi.

^b^ ++ strong growth, + moderate growth, +/- weak growth, Tr. trace of growth, - no growth.

^c^ ++ strong fluorescence, + moderate fluorescence, +/- weak fluorescence, Tr. trace of fluorescence, - no fluorescence.

## 4. References

1. J. Rosevear and J. F. K. Wilshire, *Aust. J. Chem.*, 1981, **34**, 839–853.

2. C. Blanchard, C. Montginoul, E. Torreilles, and L. Giral, *J. Heterocycl. Chem.*, 1981, **18**, 841–3.

3. K. Lehmstedt and H. Hundertmark, *Berichte der Dtsch. Chem. Gesellschaft*, 1931, **64**, 2386–2394.

4. D. Hodgeman and R. Prager, *Aust. J. Chem.*, 1972, **25**, 191–9.

# S1 Appendix

^1^H NMR spectrum of compound **7a.**

# S2 Appendix

^13^C NMR spectrum of compound **7a**.

# S3 Appendix

^1^H NMR spectrum of compound **7b**.

# S4 Appendix

^13^C NMR spectrum of compound **7b**.

# S5 Appendix

^1^H NMR spectrum of compound **7c**.

# S6 Appendix

^13^C NMR spectrum of compound **7c**.

# S7 Appendix

^1^H NMR spectrum of compound **9**.

# S8 Appendix

^13^C NMR spectrum of compound **9**.

# S9 Appendix

^1^H NMR spectrum of compound **11**.

# S10 Appendix

^13^C NMR spectrum of compound **11**.
